# Mutations of Kinases and GTPases in Cancers

**DOI:** 10.3390/cancers18132033

**Published:** 2026-06-23

**Authors:** Jonas Cicenas, Ramojus Balevičius, Rytė Bagdanavičiūtė, Jokūbas Šimkus

**Affiliations:** 1 Faculty of Informatics, Engineering and Technologies, Kauno Kolegija Higher Education Institution, LT-50468 Kaunas, Lithuania; 2 SMK College of Applied Sciences, Kalvarijų g. 137E, LT-08248 Vilnius, Lithuania; 3 UAB CDKjc, Kukučių g. 39, LT-51338 Kaunas, Lithuania; 4 Institute of Biochemistry, Life Sciences Center, Vilnius University, Saulėtekio al. 7, LT-102587 Vilnius, Lithuania; ramojusbalevicius@gmail.com; 5 Faculty of Medicine, Lithuanian University of Health Sciences, A. Mickevičiaus g. 9, LT-44307 Kaunas, Lithuania; 6 Vilnius University Hospital Santariškių Klinikos, Santariškių g. 2, LT-08406 Vilnius, Lithuania; jo.simkus@gmail.com

**Keywords:** protein kinases, inositol polyphosphate kinases, GTPases, cancers, leukemias, mutations

## Abstract

Cancer is a genetic disease driven by the accumulation of mutations that disrupt normal cellular growth. Among the most frequently mutated families are protein kinases, inositol polyphosphate kinases, and GTPases, which together function as central molecular switches controlling proliferation, survival, and metabolism. In cancer, activating mutations in protein kinases, such as EGFR and BRAF, lead to uncontrolled downstream signaling by locking these enzymes in a constitutively active state. Similarly, mutations affecting inositol kinases, notably PI3KCA, hyperactivate the PI3K/AKT pathway, promoting relentless cell survival and resistance to apoptosis. GTPases, particularly Ras family members (KRAS, NRAS, HRAS), are classical oncogenes where single amino acid substitutions impair their intrinsic GTP hydrolysis activity, trapping them in a persistently GTP-bound “on” state. This unleashes continuous mitogenic signaling independently of external growth factors. Collectively, these mutations are not random but converge on a limited set of core pathways, making them key drivers of tumor initiation and progression. Understanding the specific molecular consequences of kinase and GTPase mutations has directly informed the development of targeted therapies, including small molecule inhibitors and monoclonal antibodies, now used in routine clinical practice.

## 1. Introduction

Cancer encompasses a spectrum of disorders characterized by the uncontrolled growth and dissemination of abnormal cells. Both extrinsic factors—including chemical carcinogens, infectious agents, and poor dietary habits—and intrinsic defects—such as inherited gene mutations, hormonal imbalances, or immune dysfunctions—can initiate malignant transformation. In the United States alone, over 2.1 million new cancer diagnoses are projected for 2026, with approximately 625,000 Americans expected to succumb to the disease. Across the European Union, annual cancer deaths are estimated to be 2.7 million in 2026, with approximately 1.2 deaths. Among the most lethal malignancies worldwide are cancers of the breast, colorectum, lung, pancreas, and prostate, along with melanomas and leukemias.

Protein kinases constitute a large enzyme family responsible for phosphorylating proteins at specific amino acid residues. They are divided into two major subfamilies: tyrosine kinases and serine/threonine kinases. The human genome encodes 518 protein kinases, of which 478 possess a canonical protein kinase domain and 40 are classified as atypical kinases. By mediating protein phosphorylation, kinases regulate a broad range of cellular processes, including proliferation, growth, cell cycle progression, differentiation, survival, and apoptosis. Disruption of normal kinase activity can profoundly alter these pathways and contribute to numerous diseases, notably cancers, but also neurodegenerative disorders, inflammatory conditions, diabetes, kidney diseases, and cardiovascular ailments [1]. Well-known oncogenes frequently encode proteins belonging to the kinase, GTPase, transcription factor, or growth factor families. In this review, we concentrate on two Ras superfamily members (KRAS and NRAS) and one protein kinase superfamily member (BRAF).

The Ras superfamily, also referred to as small GTPases, comprises low-molecular-weight proteins (20–25 kDa) that share the ability to bind guanosine triphosphate (GTP) and diphosphate (GDP). GTPases are active in the GTP-bound state and inactive when bound to GDP; replacement of GDP with free GTP restores activity, providing an on–off switching mechanism. Over 160 Ras proteins have been identified in humans [2]. Historically, RAS genes were discovered through studies of sarcoma-inducing viruses in rats, giving rise to the name “Rat sarcoma.” The first three-dimensional structure of a Ras protein was solved in 1990 [3], and over 450 structures from higher eukaryotes, yeasts, bacteria, and archaea are now deposited in the RCSB Protein Data Bank. Ras proteins participate in numerous critical cellular processes, including cell cycle control, survival signaling, actin cytoskeleton organization, and nuclear and vesicular transport [2]. This editorial will analyze some examples of well known mutations of kinases and GTPases.

## 2. Examples Mutations of Kinases and GTPases

### 2.1. EGFR

**Figure 1:**
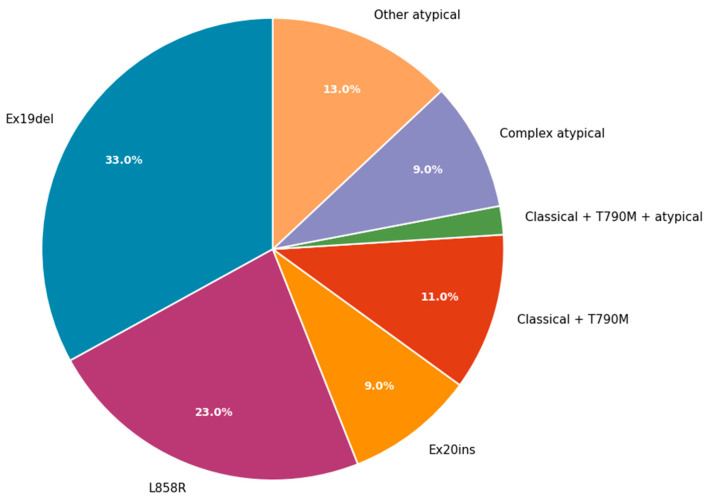
EGFR mutation subtypes in non-small cell lung cancer.

The epidermal growth factor receptor (EGFR) is a transmembrane receptor tyrosine kinase belonging to the ErbB family, which plays a central role in regulating cellular proliferation, differentiation, and survival upon binding to its cognate ligands such as EGF and TGFα. Upon ligand-induced activation, EGFR undergoes homodimerization or heterodimerization with other ErbB family members, leading to autophosphorylation of intracellular tyrosine residues and subsequent activation of downstream signaling cascades including the RAS-RAF-MEK-ERK and PI3K-AKT pathways. Dysregulation of EGFR through gene amplification, overexpression, or activating mutations results in sustained mitogenic signaling and is frequently implicated in the pathogenesis of multiple human cancers, most notably non-small cell lung cancer (Figure 1), colorectal cancer, and glioblastoma. A study in non-small cell lung cancer (NSCLC) demonstrated that texture analysis from enhanced MRI and pathological slides can reliably predict EGFR mutation status in breast cancer, achieving an AUC of 0.983, an accuracy of 96.2%, and a sensitivity of 97.9% [4]. In metastatic colorectal cancer, it was highlighted that EGFR extracellular domain mutations such as S492R and R451C prevent antibody binding while preserving receptor kinase activity, representing key mediators of acquired resistance to anti-EGFR therapy [5]. Ali and colleagues reported that EGFR exon 20 mutations are associated with significantly higher SUVmax values on [^18^F]FDG PET-CT imaging in metastatic colorectal cancer, with SUVmax demonstrating potential as a non-invasive predictive tool for determining EGFR mutation status [6]. Jeon et al. identified the rare EGFR L833V/H835L cis compound mutation in 8 of 3835 NSCLC patients screened (0.21% prevalence) and confirmed that this mutant exhibits oncogenic potential while remaining sensitive to EGFR tyrosine kinase inhibitors [7]. An Austrian real-world study of 1267 advanced NSCLC patients reported an EGFR mutation prevalence of 11% (145 patients); among 53 patients with stage IIIB or higher disease, median overall survival was 17.7 months, with exon 19 deletion carriers achieving the longest median overall survival (32.5 months) compared to L858R mutation carriers (17 months) [8]. A separate international real-world study of 1043 resected early-stage NSCLC patients found EGFR mutations in 32% of cases, with five-year overall survival rates of 84% for mutation-positive patients versus 64% for mutation-negative patients [9]. Resistance mechanisms to EGFR-TKIs revealed that while osimertinib overcomes the T790M mutation, emergent C797S mutations and MET amplifications subsequently compromise its efficacy [10]. Wenzhou Medical University reported a case of NSCLC harboring both an EGFR exon 19 deletion and the T790M missense mutation at baseline, where the patient achieved 8 months of progression-free survival on furmonertinib before developing breast metastasis, illustrating that coexisting sensitive and resistant mutations negatively affect TKI efficacy [11]. In a retrospective study of 2800 EGFR-mutant NSCLC patients, 9 patients (0.32%) who developed acquired ALK, RET, or ROS1 fusions following TKI therapy were identified, with subsequent fusion-targeted therapy yielding progression-free survival ranging from 5 to 39 months [12]. Interestingly, German researchers estimated that RET fusions occur as an acquired resistance mechanism in 0.2% of EGFR-mutated patients progressing on TKIs. In these cases, combined EGFR and RET inhibition produced progression-free survival of 3.9 to 13.0 months, and personalized droplet digital PCR enabled non-invasive longitudinal monitoring of resistance dynamics [13].

### 2.2. MET

The mesenchymal–epithelial transition factor (MET) is a receptor tyrosine kinase encoded by the MET proto-oncogene. Upon binding of its cognate ligand hepatocyte growth factor (HGF), MET activates downstream signaling cascades, including RAS-ERK and PI3K-AKT pathways, that regulate cell proliferation, survival, migration, and morphogenesis. Physiological MET signaling is tightly regulated through autophosphorylation and ubiquitin-mediated degradation; however, genetic alterations such as activating mutations, gene amplification, or exon 14 skipping can lead to constitutive receptor activation and oncogenic transformation. Dysregulated MET signaling has been implicated in multiple cancer types, most notably non-small cell lung cancer (NSCLC), where MET exon 14 skipping mutations represent a clinically actionable driver alteration, as well as in hereditary papillary renal cancer, gastric cancer, and glioblastoma. A single-center real-world study analyzed 1374 lung cancer patients without EGFR or ALK mutations and identified MET exon 14 skipping mutations in 69 patients (5.0% of this selected population), with a notably higher frequency observed in pulmonary sarcomatoid carcinoma (24.3%, 9 of 37 patients) [14]. The results showed that among 29 stage IV lung adenocarcinoma patients with MET exon 14 skipping who received active treatment, pemetrexed-based chemotherapy (*p* = 0.003), lung radiotherapy (*p* = 0.020), initial brain metastasis (*p* = 0.005), and strong c-MET immunohistochemical staining (*p* = 0.012) were independent prognostic factors for overall survival, whereas stage IV pulmonary sarcomatoid carcinoma patients showed similarly poor outcomes regardless of MET mutation status [14]. A Chinese study of 17 NSCLC patients with MET exon 14 skipping mutations found that 9 patients (53%) harbored MET amplification and 10 patients (59%) exhibited MET overexpression, with a significant correlation between these two alterations (Pearson’s r^2^ = 0.4657, *p* < 0.005), although neither MET amplification nor overexpression correlated with clinicopathological characteristics or overall survival [15]. An analysis of germline mutations in an oncologic cohort of 25,220 patients with cancer revealed that MET germline mutations were present in only one patient (0.004%), and this individual was diagnosed with renal cell carcinoma [16]. A comprehensive review of MET mutations in cancers traced the discovery from the first MET mutation in hereditary papillary renal cancer in 1997 to the identification of MET exon 14 skipping mutations in lung cancer in 2014, noting that less than five years after their discovery, MET tyrosine kinase inhibitors demonstrated efficacy in NSCLC patients harboring these alterations, although acquired resistance mutations subsequently emerged [17]. A scoping review of 159,993 Chinese NSCLC patients reported that the frequency of MET exon 14 skipping mutations ranged from 0.08% to 1.38% of all NSCLC patients and from 8.33% to 56.60% of all MET mutations, highlighting the marked heterogeneity across studies [18]. A three-year screening experience in France involving 1143 consecutive NSCLC patients identified 46 MET exon 14 skipping alterations (4.0% prevalence), of which 4 were not detected by DNA sequencing alone and required fragment analysis or RNA sequencing [19]. The response to MET inhibitors (crizotinib or capmatinib) evaluated in 15 patients yielded an objective response rate of 44% with a median progression-free survival of 5.5 months, while 13 patients treated with immunotherapy achieved an objective response rate of 30% and median progression-free survival of 4.0 months, with no correlation observed between response and PD-L1 expression [19]. A pan-cancer analysis of 4149 patients across 12 tumor types treated with immune checkpoint inhibitors demonstrated that MET mutation was associated with favorable overall survival (hazard ratio 0.61), progression-free survival (hazard ratio 0.74), and objective response rate (40.3% vs. 28.1% in MET wild-type), with multi-omics analysis revealing enhanced tumor immunogenicity and enriched immune cell infiltration in MET-mutant tumors [20]. A large Chinese next-generation sequencing study of 11,330 lung cancer patients detected MET exon 14 skipping mutations in 2.20% of cases using synchronous DNA- and RNA-based approaches, identifying 45 distinct variants distributed across a 197-base pair DNA sequence [21]. The incidence of MET exon 14 skipping was significantly higher in patients aged 60 years and above (*p* < 0.0001) and in needle biopsy samples (*p* < 0.0001), with a positive correlation observed between microsatellite instability-high status and MET exon 14 skipping (*p* < 0.0001) [21]. An analysis of the immune microenvironment in nine NSCLC patients with MET exon 14 skipping mutations using multiplex immunofluorescence revealed that CD8+TIM3+ and CD8+LAG3+ cells were predominantly localized in the tumor parenchyma in recurrent/metastatic patients but in the stroma in non-recurrent patients, with Cox regression analysis suggesting a potential association between higher densities of CD8+TIM3+ cells and improved disease-free survival (hazard ratio 0.89) [22]. A large Chinese cohort study of 30,355 lung cancer and 6004 brain tumor patients found that clinically significant MET mutations were more frequent in lung cancer (*p* < 0.001) [23]. MET alterations were significantly enriched in post-treatment brain tumors compared to treatment-naïve tumors (8.5% vs. 4.8%, *p* < 0.001), and while MET mutations were not prognostic in lung cancer, they were associated with significantly poorer survival in brain tumors (median overall survival 19.9 vs. 62.9 months, *p* < 0.001), underscoring that the clinical significance of MET alterations is highly context-dependent [23]. A case report from Japan described a lung cancer patient with MET exon 14 skipping mutation presenting with a characteristic “crazy-paving” appearance on chest imaging, highlighting a potential radiologic clue for this molecular subtype [24].

### 2.3. FLT

FMS-like tyrosine kinase 3 (FLT3) is a class III receptor tyrosine kinase that plays a critical role in hematopoietic progenitor cell proliferation, differentiation, and survival, primarily through activation of the RAS-MAPK, STAT5, and PI3K-AKT signaling pathways. Upon binding of its cognate ligand, FLT3 undergoes dimerization and autophosphorylation, leading to downstream activation of transcriptional programs that regulate normal hematopoiesis; however, activating mutations such as internal tandem duplications (ITDs) and tyrosine kinase domain (TKD) point mutations result in constitutive receptor signaling and are among the most common genetic alterations in acute myeloid leukemia (AML). Beyond hematologic malignancies, FLT3 has also been implicated in solid tumors; for example, FLT3 was identified as a potential prognostic biomarker for favorable outcomes in pancreatic ductal adenocarcinoma, where higher FLT3 levels correlated with improved patient survival and may guide future treatment selection [25]. An acute myeloid leukemia study examined FLT3 mutations in 65 newly diagnosed AML patients and found FLT3-ITD frequencies of 16.9% and FLT3-TKD frequencies of 10.8%, with one patient harboring both mutation types [26]. White blood cell counts and peripheral blast percentages were significantly higher in FLT3-ITD-positive patients, while FLT3-TKD-positive patients had significantly lower peripheral blast percentages. DNA sequencing identified four distinct TKD mutations, including the pathogenic Asp835Val and Asp835His alterations [26]. A Syrian study of 100 adult AML patients with normal karyotype reported a FLT3-ITD prevalence of 24%, including four patients with novel duplication patterns not previously described, and demonstrated that FLT3-ITD positivity was significantly associated with elevated lactate dehydrogenase levels, poor overall survival, higher relapse rates, and shorter event-free survival [27]. The frequency of FLT3-TKD mutations in the Syrian cohort was low at 2%, and no compound ITD/TKD mutations were detected [27]. An analysis of 45 AML patients with FLT3-ITD mutations revealed that 13% carried multiple FLT3-ITD clones, and mutations were classified as duplication-only (52%) or duplication with insertions (48%) [28]. The duplication-with-insertion variant was independently associated with poor prognosis among non-APL patients (odds ratio of 2.92), similar to FLT3-ITD with a variant allele frequency of 50% or higher, highlighting that the specific mutation type carries important prognostic value beyond simple ITD detection [28]. A pediatric and young adult study of 464 FLT3-ITD-positive AML patients treated on Children’s Oncology Group trials found that 79% had co-occurring mutations across 239 different genes, and patients with co-occurring favorable-risk mutations (NPM1, CEBPA, t(8;21), or inv(16)) experienced a 5-year event-free survival of 64%, compared to only 22.2% for those with poor-risk mutations (WT1, UBTF, or NUP98::NSD1) [29]. Multivariable analysis demonstrated that the co-occurring mutational profile had significant prognostic impact, whereas the FLT3-ITD allelic ratio had no impact, indicating that risk stratification and therapeutic allocation in pediatric FLT3-ITD AML should be guided primarily by cooperating mutation status rather than ITD burden [29]. An analysis of 63 de novo AML patients identified FLT3-ITD mutations in 22% of cases and discovered a novel mutation in the juxtamembrane domain (exon 14) in 6 patients (10%), which resulted in a tyrosine-to-valine substitution at amino acid 572 and created a premature stop codon [30]. In silico modeling predicted that this novel mutation leads to loss of most of the juxtamembrane domain and the entire kinase domain, likely affecting FLT3 receptor activity and warranting further functional characterization [30]. Real-world data from the Thai Acute Leukemia Working Group encompassing 360 adult AML patients from 11 institutions nationwide reported a FLT3-ITD prevalence of 28.1%, with FLT3-ITD-positive patients showing higher white blood cell counts, higher bone marrow blast percentages, and more frequent NPM1 co-mutations compared to FLT3-wild-type patients [31]. Complete remission was achieved in 55.7% of FLT3-ITD patients versus 66.5% of FLT3-wild-type patients, and median overall survival was significantly shorter in the FLT3-ITD group (8.8 vs. 13.2 months, *p* = 0.039), with multivariable analysis confirming FLT3-ITD as an independent predictor of poor overall survival [31]. A case report described a 72-year-old AML patient harboring the rare non-canonical FLT3 V491L mutation, and ex vivo sensitivity testing demonstrated that gilteritinib had the lowest half-maximal inhibitory concentration (170.6 nM) compared to midostaurin (547.8 nM) and quizartinib (800.6 nM), correlating with clinical improvement observed during gilteritinib treatment [32]. A large real-world genomic study of 1151 de novo AML patients who received intensive chemotherapy found FLT3-ITD in 19.6% of cases, FLT3-TKD in 5.6% of cases, co-occurring ITD and TKD in 1.3%, and non-canonical FLT3 mutations in 1.1% of cases [33]. Among patients receiving FLT3 inhibitors, complete remission rates were significantly higher (94.7% vs. 71.5%), relapse rates were lower (37.7% vs. 58.5%), and overall survival significantly improved (median 59 vs. 17 months), with FLT3 inhibitor treatment independently associated with improved survival in multivariable analysis (hazard ratio of 0.39) [33]. Transcriptomic profiling of FLT3-ITD AML revealed significant enrichment of metabolic pathways including oxidative phosphorylation, DNA repair, and fatty acid metabolism, alongside suppression of heme metabolism and immune-related programs (interferon alpha, interferon gamma, and inflammatory responses), a signature that was externally validated in an independent Beat AML cohort [33].

### 2.4. BRAF

BRAF is a serine/threonine protein kinase that functions as a critical downstream effector of the RAS-RAF-MEK-ERK signaling cascade, transmitting mitogenic signals from the cell surface to the nucleus to regulate cell proliferation, differentiation, and survival. The most common activating alteration, BRAF V600E, results from a thymine-to-adenine transversion at codon 1799, leading to a valine-to-glutamic acid substitution that confers constitutive kinase activity independent of upstream RAS activation. BRAF mutations are prevalent in multiple cancer types, including melanoma (40–60%), thyroid cancer (30–80%), colorectal cancer (5–15%), and non-small cell lung cancer (1–3%), and are associated with distinct clinicopathological features and poor prognosis in the metastatic setting. A comprehensive review on KRAS, NRAS and BRAF mutations in colorectal cancer and melanoma summarized that BRAF mutations occur in approximately 11% of colorectal cancers and 50–60% of cutaneous melanomas, with the V600E substitution being the most common alteration in both tumor types [34]. A case report described a 60-year-old woman with triple-negative breast cancer harboring a BRAF V600E mutation who received vemurafenib plus albumin-bound paclitaxel as second-line therapy after progressing on first-line chemotherapy (progression-free survival of 7 months) [35]. The patient achieved 4.4 months of progression-free survival on vemurafenib-containing therapy, with regression of some pulmonary metastases but concomitant progression of other lesions, demonstrating intratumoral heterogeneity and acquired resistance mechanisms including new mutations in PDGFRB, NF2, FOXA1, and AR amplification detected upon re-biopsy of a progressed lesion [35]. The patient ultimately survived 12 months from the diagnosis of metastatic disease, leading the authors to suggest that the BRAF V600E mutation may represent a poor prognostic factor in breast cancer and a potential therapeutic target, although heterogeneous responses to BRAF inhibition remain a significant clinical challenge [35]. A study analyzed 57 epithelial ovarian tumor samples and found that the BRAF V600E mutation was present in 40% of serous borderline tumors (2 of 5 cases) and in 14.3% of mucinous borderline tumors (1 of 7 cases), but was absent in all invasive serous carcinomas (0 of 30 high-grade and 0 of 6 low-grade) and in all mucinous carcinomas (0 of 7 cases), with a statistically significant difference between borderline and invasive serous tumors (*p* = 0.017) [36]. No BRAF mutations were detected in the 57 normal ovarian tissue controls [36]. These findings support the dualistic model of ovarian carcinogenesis in which borderline tumors and high-grade invasive carcinomas develop via distinct molecular pathways, with BRAF mutations characterizing the former but not the latter [36]. A study of 219 patients with hairy cell leukemia identified 20 patients (9.1%) harboring non-V600E BRAF mutations, including 14 with a single non-V600E mutation and 6 with V600E co-occurring with one or two non-V600E alterations [37]. The most prevalent non-V600E alteration was a deletion in exon 12 (N486_P490del or V487_T491del) observed in 10 patients, all without V600E, while the activation loop mutations V600D and S607P were also detected [37]. Patients with non-V600E BRAF mutations treated with purine analog monotherapy had a median relapse-free survival of only 20.4 months, significantly inferior to the 260 months observed in classic hairy cell leukemia controls (*p* < 0.0001), whereas the incorporation of rituximab into first-line therapy improved median relapse-free survival to not reached (*p* = 0.0023) [37]. A comprehensive review of BRAF alterations in chronic lymphocytic leukemia (CLL) synthesized data from multiple published cohorts and reported that BRAF mutations are detected in approximately 2–6% of unselected CLL patients, with higher frequencies observed in biologically enriched or treatment-selected populations, and that non-V600E variants predominate when broad sequencing is used [38]. In chemoimmunotherapy-dominant cohorts, BRAF mutations were associated with earlier time to first treatment (e.g., 2 vs. 23 months in one IGH-translocated subset) but not consistently with inferior overall survival, whereas in targeted-therapy-era studies, BRAF alterations recurred as part of MAPK-pathway-driven clonal evolution at relapse after BTK, PI3K, or BCL2 inhibitors [38]. The review concluded that BRAF-mutated CLL should be interpreted within the broader RAS-RAF-MAPK-ERK signaling context rather than as a classical V600E-driven entity, with clinical relevance greatest in trisomy 12-enriched disease, genomically complex cases, Richter transformation with V600E lesions, and treatment-exposed relapse [38]. A case report described a 70-year-old male with a history of chronic lymphocytic leukemia and recurrent stage IIIC melanoma whose molecular testing yielded discordant results, with one commercial laboratory reporting a BRAF V600E mutation while two separate NGS assays (performed at initial diagnosis and at the authors’ institution) consistently demonstrated an NRAS G12D mutation and absence of BRAF V600E [39]. Given that NRAS and BRAF mutations are mutually exclusive in 99.4% of melanomas, and repeat testing using both single-gene Sanger sequencing and comprehensive NGS with a germline control confirmed BRAF wild-type status, the BRAF V600E result was deemed a false positive [39]. The patient was treated with neoadjuvant ipilimumab and nivolumab according to the NADINA trial, achieving a complete radiographic response after one cycle and a pathologic partial response upon surgical resection, highlighting the importance of confirming discordant mutational results with secondary technology before initiating targeted therapy [39]. A large retrospective cohort study analyzed 2446 consecutive colorectal cancer patients and identified 146 BRAF-mutant cases (5.97% overall prevalence), which was significantly lower than the 11.8–16.3% reported in Western cohorts (adjusted odds ratio of 0.48, *p* = 0.004) [40]. Among BRAF-mutant patients, the V600E subtype accounted for 54.1% of cases. These patients were younger at diagnosis, had fewer metastatic cases at presentation, and exhibited distinct co-mutation patterns including a significantly higher prevalence of TP53 co-mutations (66% vs. 32% in Western cohorts, *p* < 0.001), which were associated with poor prognosis [40]. The BRAF V600E subtype was associated with significantly worse overall survival (*p* = 0.0036), increased risk of peritoneal metastasis (*p* < 0.001), female sex, advanced disease stage, and poor tumor differentiation compared to non-V600E patients, while non-V600E tumors exhibited a higher tumor mutational burden (*p* < 0.05) [40]. In terms of treatment response, patients with BRAF V600E mutations demonstrated markedly inferior outcomes to first-line therapy compared to non-V600E cases (objective response rate of 21.1% vs. 63.2%, *p* < 0.01), particularly in those receiving bevacizumab-based regimens where median progression-free survival was 5.2 months versus 9.1 months (*p* = 0.0025) [40]. A multi-institutional study of 97 BRAF-mutant non-small cell lung cancer (NSCLC) patients, supplemented by an independent clinico-genomic database of 342 patients, demonstrated that patients with class I BRAF mutations (primarily V600E) treated with BRAF-MEK inhibitors at any line had significantly longer median overall survival compared to those who did not receive these agents (40 vs. 10 months, *p* = 0.043), whereas no significant survival difference was observed between those treated with immune checkpoint inhibitors versus those not treated [41]. Among class I patients, 54% had PD-L1 expression of 50% or higher, yet immunotherapy did not confer a survival advantage (30 vs. 46 months, *p* = 0.908), supporting first-line targeted therapy over immunotherapy in this molecular subset [41]. Tumors with class II or III BRAF variants were significantly more likely to harbor concurrent MAPK pathway alterations relative to class I (39.2% and 25.8% vs. 7.1%, *p* < 0.0001), and cell line studies identified genetic dependency on BRAF in class II cells without sensitivity to BRAF inhibitors, while class III cells showed dependency on EGFR, suggesting distinct therapeutic vulnerabilities for atypical BRAF mutation classes [41]. A large real-world study of 161 patients with BRAF V600E-mutated metastatic colorectal cancer reported a median age of 58.5 years; 59.6% were female, 70.8% had right-sided primary tumors, and 21.7% exhibited deficient mismatch repair [42]. With a median follow-up of 23 months, first-line progression-free survival was 9 months. Only 26% of patients received BRAF inhibitors in the second-line setting, achieving a median progression-free survival of 5.2 months and an objective response rate of 10.5%, reflecting significant barriers to access to high-cost targeted therapies [42]. A study of 26 patients with unresectable BRAF V600E-mutated colorectal cancer reported a median overall survival of 12.0 months, with right-sided tumors present in 46.2% of cases and poorly differentiated or mucinous adenocarcinoma in 30.8% of cases [43]. Patients treated with the BEACON regimen (encorafenib plus cetuximab with or without binimetinib) achieved a median overall survival of 13.3 months, which was significantly better than those treated without this regimen (7.2 months; hazard ratio of 4.180, *p* = 0.029), with a median progression-free survival of 6.6 months for BEACON-treated patients [43]. A study of 10,211 lung cancer patients identified 88 patients with primary BRAF mutations and 15 patients with acquired BRAF mutations arising as a resistance mechanism after EGFR tyrosine kinase inhibitor therapy [44]. Primary BRAF-mutated patients were more frequently elderly (median age 67 vs. 61 years, *p* = 0.015), male (53.4% vs. 26.7%, *p* = 0.056), and former or current smokers (36.5% vs. 6.7%, *p* = 0.033) compared to acquired BRAF-mutated patients [44]. For primary BRAF/EGFR co-mutated patients with non-exon 19 deletions, the addition of dabrafenib and trametinib to third-generation EGFR tyrosine kinase inhibitors achieved an objective response rate of 100% (3 of 3 patients) and a median progression-free survival of 12 months, while acquired BRAF/EGFR co-mutated patients receiving triple-targeted therapy achieved a median progression-free survival of 8.6 months [44]. A large cohort analysis of 5937 patients with stage I-IV colon cancer from a national database (2004–2020) found that 31.4% of tested tumors harbored BRAF mutations (reflecting testing selection bias), with BRAF mutation strongly associated with microsatellite instability (OR = 6.66, *p* < 0.001), female sex, older age, right-sided tumors, and poor differentiation [45]. In multivariable analysis, BRAF mutation was independently associated with worse overall survival across all stages (HR = 1.35, *p* < 0.001), but this effect was limited to stage IV disease (HR = 1.45, *p* < 0.001) with no significant survival difference in stages I–III [45]. When stratified by MSI status, BRAF mutation conferred worse survival in microsatellite-stable tumors (HR = 1.61, *p* < 0.001) but had no prognostic impact in MSI-high tumors (HR = 0.95, *p* = 0.581); moreover, among microsatellite-stable tumors, BRAF mutation was associated with higher odds of metastatic disease (OR = 1.30) and perineural invasion (OR = 1.35), whereas among MSI-high tumors it was associated with lower odds of metastasis (OR = 0.60), highlighting the biological and prognostic heterogeneity of BRAF-mutated colon cancer [45]. A descriptive study examined BRAF mutational status in 190 colorectal cancer patients and found a prevalence of 5.3% (10 patients), with a significant positive correlation observed between BRAF mutation and right-sided tumor location (*p* = 0.001) as well as microsatellite instability (*p* = 0.004) [46]. No statistically significant associations were established between BRAF mutation and age (*p* = 0.682), gender (*p* = 0.392), histological grade (*p* = 0.594), or peritoneal metastases (*p* = 0.707) [46].

### 2.5. KRAS

KRAS is a small GTPase belonging to the Ras superfamily that functions as a critical molecular switch in the RAS-RAF-MEK-ERK signaling pathway, transducing signals from cell surface receptors to the nucleus to regulate cell proliferation, differentiation, and survival. In its active state, KRAS binds GTP and interacts with downstream effectors; intrinsic GTPase activity, enhanced by GTPase-activating proteins (GAPs), hydrolyzes GTP to GDP, returning the protein to its inactive state. Activating mutations in KRAS, most commonly at codons 12, 13, and 61, impair GTP hydrolysis and lock the protein in a constitutively active conformation, leading to uncontrolled cell growth; KRAS mutations are among the most frequent oncogenic alterations in human cancers, occurring in approximately 95% of pancreatic ductal adenocarcinomas, 45% of colorectal cancers, and 35% of non-small cell lung cancers. A comprehensive review on gene mutations in pancreatic cancer summarized that KRAS is mutated in 70–95% of pancreatic cancers, with G12 codon mutations accounting for 99% of all KRAS alterations (G12D alone representing 50%), while TP53 is mutated in 20–76% of cases, CDKN2A in 49–98%, SMAD4 in 19–50%, BRCA1 in 6.6–14%, and BRCA2 in 3.6–7.5% [47]. A study developing a machine learning-based gene-mutation algorithm for predicting treatment response in breast cancer identified a novel 12-gene algorithm incorporating mutation profiles of KRAS, PIK3CA, MAP3K1, MAP2K4, PTEN, TP53, CDH1, GATA3, KMT2C, ARID1A, RUNX1, and ESR1 [48]. The algorithm distinguished non-progressed (responder) versus progressed (non-responder) patients with an AUC of 0.96 in the training cohort and 0.97 in the validation cohort and predicted progression-free survival with a hazard ratio of 21.6 (95% CI 11.3–41.5, *p* < 0.0001) in all breast cancer patients [48]. In the triple-negative breast cancer subgroup, the algorithm predicted progression-free survival with a hazard ratio of 19.3 (95% CI 3.7–101.3, *p* = 0.000) in the training cohort and 18.6 (95% CI 4.4–79.2, *p* < 0.0001) in the validation cohort, demonstrating potential to assist personalized therapies and reduce mortality in this aggressive subtype [48]. A study on B-cell acute lymphoblastic leukemia (B-ALL) found that Reh cells bearing the KRAS-G12D mutation displayed increased proliferation rates in vitro but severely compromised growth in mice, and further investigation revealed that KRAS-G12D rewired methionine and arginine metabolism, promoting catabolism of these amino acids to support anabolism of polyamines and proline, respectively [49]. Isotope tracing confirmed enhanced polyamine biosynthesis from methionine via 5′-methylthioadenosine accumulation, and chemical inhibition of polyamine biosynthesis with difluoromethylornithine (DFMO) selectively killed KRAS-G12D B-ALL cells, with significantly lower IC50 values compared to control cells [49]. The study also demonstrated that KRAS-G12D B-ALL cells displayed activated AKT/mTOR signaling, and chemical inhibition of mTOR with AZD-8055 partially rescued the growth defects of KRAS-G12D cells under nutrient-limited conditions and promoted their in vivo growth in xenograft models, suggesting that hyperactivated mTOR signaling contributes to the metabolic vulnerability induced by mutant KRAS in hematological malignancies [49]. A study investigating K-RAS mutations and mRNA expression in breast cancer found that 14% of patients harbored K-RAS mutations in codon 12 and 10% harbored K-RAS mutations in codon 13, with a significant relationship observed between K-RAS mutations and T staging as well as progesterone receptor positivity in tumors [50]. The five-year overall survival was 8% in patients with K-RAS mutations compared to 69% in those without mutations, and K-RAS mRNA expression showed a significant relationship with T and N staging and five-year survival, suggesting that both mutation status and K-RAS gene expression may be used simultaneously to estimate breast cancer prognosis [50]. A retrospective multicenter study from Turkey analyzed 101 patients with KRAS-mutant metastatic non-small cell lung cancer and found that KRAS G12C mutation was detected in 69 patients (68.3%) while KRAS non-G12C mutations were detected in 32 patients (31.7%), with G12V (14.8%) and G12D (5.9%) being the most common non-G12C subtypes [51]. The majority of patients in both groups were male (91.3% vs. 84.4%) and smokers or former smokers (92.8% vs. 90.6%), with adenocarcinoma as the predominant histology (88.4% vs. 81.3%). No statistically significant difference in PD-L1 expression was observed between the groups (21.7% vs. 34.4%, *p* = 0.132) [51]. The most common co-mutation accompanying KRAS G12C was TP53 (23%), while Rictor was the most common co-mutation in the KRAS non-G12C group (36.3%), and no significant differences were found between KRAS subtypes in terms of objective response rate to first-line treatment (47.5% vs. 48.3%, *p* = 0.657), median progression-free survival (4.46 vs. 5.23 months, *p* = 0.852), or median overall survival (14.46 vs. 15.36 months, *p* = 0.201) [51]. A large retrospective cohort study using the National Cancer Database analyzed 52,534 colorectal cancer patients with documented KRAS mutation status and found that KRAS-mutant tumors comprised 39.9% of the cohort and were more frequently right-sided and metastatic at presentation [52]. In multivariable analysis, KRAS mutation was independently associated with worse overall survival across all stages (HR 1.12), with stage-specific associations most pronounced in stage II (HR 1.14), stage III (HR 1.16), and stage IV (HR 1.07) disease [52]. In exploratory treatment-pathway analyses, KRAS mutation was associated with inferior overall survival in several common regimens, including surgery plus chemotherapy in stage III and stage IV disease, underscoring the importance of KRAS testing to optimize individualized treatment strategies [52]. A genome-wide association study in 7071 individuals with colorectal cancer investigated whether germline genetic background modifies somatic KRAS mutation frequency, followed by validation analysis in 2482 individuals [53]. No single-nucleotide variants were significantly associated with KRAS-mutant colorectal cancer (*p* < 0.0005), although one variant (rs73067863-T) showed a non-significant exploratory association with fewer KRAS-mutant tumors in the combined sample (*p* = 9.7 × 10^−7^, OR = 0.75) [53]. A Cochrane protocol outlined plans to assess the benefits and harms of G12C inhibitors (sotorasib, adagrasib) compared to chemotherapy in second-line and beyond for adults with advanced or metastatic non-small cell lung cancer harboring KRAS G12C mutations [54]. The protocol noted that KRAS is the most common oncogenic driver mutation in NSCLC, seen in 30–40% of adenocarcinoma cases in Western populations (5–15% in Asian populations), with the G12C subtype accounting for approximately 40% of all KRAS mutations [54]. A study using the AACR GENIE Biopharma Consortium Pancreas dataset analyzed 1032 pancreatic ductal adenocarcinoma patients and found that 949 (92%) exhibited mutant KRAS, predominantly at G12D (41%), G12V (32%), and G12R (16%) [55]. In patients with localized disease, those with G12V mutation had notably longer survival compared to those with the G12D mutation (*p* = 0.03), whereas patients with metastatic disease and the G12V mutation experienced shorter overall survival compared to those with G12R (*p* = 0.04) and G12D mutations (*p* = 0.04) [55]. No significant differences were observed in the frequencies of co-altered driver genes (TP53, CDKN2A, SMAD4) across different KRAS mutations, demonstrating that codon-specific KRAS mutations affect pancreatic cancer outcomes differently based on disease stage at diagnosis [55]. A study using the Japanese Center for Cancer Genomics and Advanced Therapeutics (C-CAT) database analyzed 1875 biliary tract cancer patients treated with first-line gemcitabine plus cisplatin (GC) or GC plus immune checkpoint inhibitor (ICI) and found that KRAS mutations were identified in 21.0% of patients and were significantly associated with inferior overall survival in both treatment groups [56]. KRAS mutations showed a numerically higher hazard ratio in the GC+ICI group compared to the GC group (HR 1.78 vs. 1.40), and the TP53/KRAS double-mutant subgroup demonstrated the poorest survival outcome, with shorter median overall survival on GC+ICI than on GC (11.8 vs. 15.9 months; HR 1.47), suggesting that TP53 and KRAS co-mutation may identify a molecular subset of biliary tract cancer with poor prognosis in the context of immunochemotherapy [56].

### 2.6. PIK3CA

Phosphatidylinositol-4,5-bisphosphate 3-kinase catalytic subunit alpha (PIK3CA) encodes the p110α catalytic subunit of class IA phosphoinositide 3-kinase (PI3K), which phosphorylates phosphatidylinositol (4,5)-bisphosphate to generate phosphatidylinositol (3,4,5)-trisphosphate (PIP3), a key second messenger that activates downstream effectors including AKT, mTOR, and PDK1 to regulate cell proliferation, survival, metabolism, and migration. Activating mutations in PIK3CA, most frequently occurring in the helical domain (exon 9, codons 542 and 545) and kinase domain (exon 20, codon 1047), result in constitutive PI3K signaling independent of growth factor stimulation and are among the most common oncogenic alterations in human cancers. PIK3CA mutations are found in approximately 30–40% of breast cancers, 30–50% of endometrial cancers, 15–20% of colorectal cancers, and 10–20% of cervical cancers, and have become important predictive biomarkers for PI3K inhibitor therapy. A cohort study of 1123 early-stage breast cancer patients tested for the three PIK3CA hotspot mutations (H1047R, E545K, E542K) by qPCR found an overall mutation rate of 26.7% (300 of 1123 patients), with significantly higher frequencies observed in steroid hormone receptor-positive, HER2-negative tumors (31.4%) and in grade 1 and 2 tumors (32.8%) [57]. While no significant association between PIK3CA mutations and recurrence-free interval was observed overall, a strong trend for impaired recurrence-free interval was noted in hormone receptor-positive breast cancers with PIK3CA mutations (adjusted HR 1.64), and a significantly detrimental prognostic impact was observed in hormone receptor-positive, HER2-negative patients treated with aromatase inhibitors alone (adjusted HR 4.44) but not in tamoxifen-treated patients, suggesting that estrogen deprivation may be ineffective in the presence of PIK3CA mutations [57]. A systematic review and meta-analysis of 7 studies comprising 1098 endometrial cancer patients evaluated the impact of PIK3CA exon 9 and/or exon 20 mutations on survival and found a tendency toward impaired survival for patients with PIK3CA mutations (RR 1.28; 95% CI 0.84–1.94; *p* = 0.25), with the most prominent effect observed in low-grade endometrial cancers (RR 2.04; 95% CI 0.90–4.62; *p* = 0.09) [58]. A study of 1377 advanced non-small cell lung cancer patients identified PIK3CA mutations in 57 patients (4.1%), of whom 22 had PIK3CA as the sole actionable genomic alteration and 35 had coexisting additional actionable alterations [59]. Patients with solitary PIK3CA mutations were older (median age 76 years), predominantly male (72.7%), had more squamous histology (45.5%), and included two never-smoker female adenocarcinoma patients, one of whom was treated with the PI3Kα-selective inhibitor alpelisib and achieved rapid clinical and partial radiological improvement, suggesting that PIK3CA may represent a targetable driver in a small minority of NSCLC patients [59]. A study of 405 advanced lung cancer patients analyzed PIK3CA mutations in matched circulating tumor DNA (ctDNA) and tissue samples using next-generation sequencing and found PIK3CA mutations in 5.68% of all samples (46 of 810), with detection rates of 5.19% in plasma and 6.17% in matched tissues [60]. The most common mutation types were p.Glu542Lys, p.Glu545Lys, and p.His1047Arg, and the concordance between ctDNA and matched tissues was 97.53% (kappa 0.770, *p* = 0.000), with a sensitivity of 72.0% and specificity of 99.2%, suggesting that ctDNA may serve as an alternative for tissue-based PIK3CA mutation detection in lung cancer [60]. A study analyzing 49,051 colorectal cancer patients from the Foundation Medicine database identified that multi-hit PIK3CA mutations (two or more activating mutations on the same allele) were present in 1.7% of patients (710 of 41,154), with the four most common PIK3CA variants being H1047R (9.8%), E545K (9.2%), E542K (9.0%), and R88Q (7.1%), and the most common variant pair was E542K-E545K (4.7%) [61]. Among patients with multi-hit PIK3CA mutations, 64.7% had co-occurring KRAS mutations and 9.1% had BRAF V600E mutations, and 17.6% exhibited microsatellite instability-high status, suggesting that multi-hit mutations may represent a subset of patients with enhanced sensitivity to PI3K inhibition [61]. A study of 97 endometrial cancer patients found that PIK3CA mutations were identified in approximately 48.5% of cases (47 of 97 patients), and the rate of lymph node metastasis was significantly higher in patients with PIK3CA mutations compared to those with wild-type PIK3CA (21.3% vs. 6.0%, *p* = 0.027) [62]. Multivariate logistic analysis revealed that PIK3CA mutation was an independent risk factor for lymph node metastasis (OR 8.58; 95% CI 1.51–48.84; *p* = 0.015), along with histological subtype II (OR 5.51) and lymphovascular space invasion (OR 7.96), and the combination of clinicopathological parameters and PIK3CA mutations more accurately predicted lymph node metastasis (AUC 0.824) [62]. A study of 48 Indian head and neck squamous cell carcinoma patients screened for the PIK3CA H1047R somatic mutation using PCR-RFLP found that 25 patients (52%) carried a heterozygous form of the mutation (His/Arg) while the remaining 23 (48%) were wild type (His/His), with no homozygous mutations detected [63]. The mean overall survival of patients with the H1047R mutation was 20.5 months compared to 26.3 months in wild-type patients, and the mean progression-free survival was 18.6 months in mutation carriers versus 26.3 months in wild-type patients, suggesting that Indian HNSCC patients with PIK3CA H1047R mutation have poor prognosis [63]. A study of 414 South Indian women (204 cervical cancer cases and 210 controls) analyzed mutations in PIK3CA, KRAS, and PTEN genes and found mutation frequencies of 16.66% for PIK3CA, 6.37% for KRAS, and 2.45% for PTEN in cervical cancer cases, while HPV infection was detected in 87.10% of cervical cancer patients [64]. A study of 448 non-metastatic colorectal cancer patients from The Cancer Genome Atlas (TCGA) and 655 non-metastatic colorectal cancer patients from an institutional cohort found PIK3CA mutation rates of 26.3% in the TCGA cohort and 7.8% in the institutional cohort, with mutations significantly associated with tumor site, TNM stage, and regional lymph node metastasis [65]. Exon 9 mutations correlated with tumor site, while exon 20 mutations were linked to tumor site and lymph node metastasis, but neither PIK3CA mutations nor common exon mutations were associated with overall survival in non-metastatic colorectal cancer patients [65] (Table 1).

## 3. Conclusions

The accumulating evidence reviewed here confirms that mutations in protein kinases, inositol polyphosphate kinases, and small GTPases are not random events but converge on a limited set of core signaling pathways—EGFR, MET, FLT3, BRAF, KRAS, and PI3K—making them key drivers of tumor initiation, progression, and therapeutic resistance across diverse malignancies. The clinical impact of these mutations is highly context-dependent, with specific alterations such as EGFR exon 19 deletions, MET exon 14 skipping, FLT3-ITD, BRAF V600E, KRAS G12C, and PIK3CA H1047R each demonstrating distinct prognostic and predictive implications that vary by cancer type, disease stage, and co-mutation status. The development of targeted therapies has been particularly successful for several of these drivers, including EGFR tyrosine kinase inhibitors in non-small cell lung cancer, FLT3 inhibitors in acute myeloid leukemia, and BRAF/MEK inhibitors in melanoma and colorectal cancer. Perhaps most notably, KRAS—long considered “undruggable”—has seen the FDA approval of first-generation KRAS G12C inhibitors (sotorasib and adagrasib) for the treatment of non-small cell lung cancer and colorectal cancer, representing a landmark achievement in precision oncology [66]. While these approvals are a major step forward, ongoing pharmacovigilance studies continue to characterize the safety profiles of these agents, identifying adverse events such as hepatotoxicity, pneumonitis, and gastrointestinal toxicities that require careful clinical management [66]. Despite these advances, the emergence of acquired resistance remains a persistent challenge, driven by on-target secondary mutations, bypass pathway activation, and histologic transformation, underscoring the need for next-generation inhibitors and rational combination strategies. Beyond the six genes highlighted in this review, numerous other mutated kinases and GTPases—including KIT, RET, NRAS, HRAS, JAK2, PTPN11, and NF1—play critical roles in oncogenesis and represent important targets for future investigation. The complexity and heterogeneity of kinase and GTPase-driven cancers call for integrated genomic, transcriptomic, and proteomic approaches to fully capture the mutational landscape and inform personalized treatment decisions. We hope that this editorial will inspire researchers to explore not only mutational drivers but also the emerging role of small non-coding RNAs in regulating these essential signaling networks.

## Figures and Tables

**Table 1:** Frequency of gene mutations across cancer types.

Gene	Cancer	Frequency (%)	References
EGFR	Non-small cell lung cancer	0.2–32	[7,8,9,12,13]
MET	Non-small cell lung cancer (exon 14 skipping)	0.08–4.0	[18,19,21]
MET	Renal cell carcinoma (germline)	0.004	[16]
FLT3	Acute myeloid leukemia (ITD)	16.9–28.1	[26,27,31,33]
FLT3	Acute myeloid leukemia (TKD)	5.6–10.8	[26,33]
FLT3	Acute myeloid leukemia (novel juxtamembrane)	10	[30]
BRAF	Colorectal cancer	5.3–31.4	[40,42,43,45,46]
BRAF	Melanoma (cutaneous)	50–60	[34]
BRAF	Hairy cell leukemia (non-V600E)	9.1	[37]
BRAF	Chronic lymphocytic leukemia	2–6	[38]
BRAF	Ovarian cancer (borderline tumors)	14.3–40	[36]
BRAF	Non-small cell lung cancer (primary)	0.86	[44]
KRAS	Pancreatic cancer	70–95	[47]
KRAS	Colorectal cancer	5–39.9	[34,52]
KRAS	Non-small cell lung cancer (G12C among KRAS-mutant)	68.3	[51]
KRAS	Non-small cell lung cancer (G12C in adenocarcinoma)	5–40	[54]
KRAS	Breast cancer	10–14	[50]
KRAS	Biliary tract cancer	21	[56]
PIK3CA	Breast cancer (early-stage)	26.7	[57]
PIK3CA	Endometrial cancer	48.5	[58,62]
PIK3CA	Colorectal cancer	1.7–26.3	[61,65]
PIK3CA	Non-small cell lung cancer	4.1–6.17	[59,60]
PIK3CA	Cervical cancer	16.66	[64]
PIK3CA	Head and neck squamous cell carcinoma (H1047R)	52	[63]

## Abbreviations

The following abbreviations are used in this manuscript:

**Abbreviation**	**Full Form**
ALL	Acute lymphoblastic leukemia
AML	Acute myeloid leukemia
AUC	Area under the curve
BRAF	B-Raf proto-oncogene, serine/threonine kinase
CLL	Chronic lymphocytic leukemia
ctDNA	Circulating tumor DNA
DFMO	Difluoromethylornithine
EGFR	Epidermal growth factor receptor
ERK	Extracellular signal-regulated kinase
FDA	Food and Drug Administration
FLT3	FMS-like tyrosine kinase 3
GC	Gemcitabine plus cisplatin
GTPase	Guanosine triphosphatase
HER2	Human epidermal growth factor receptor 2
HGF	Hepatocyte growth factor
HNSCC	Head and neck squamous cell carcinoma
HPV	Human papillomavirus
HR	Hazard ratio
ICI	Immune checkpoint inhibitor
ITD	Internal tandem duplication
KRAS	Kirsten rat sarcoma viral oncogene homolog
MAPK	Mitogen-activated protein kinase
MEK	Mitogen-activated protein kinase kinase
MET	Mesenchymal–epithelial transition factor
MSI	Microsatellite instability
MSS	Microsatellite stable
mTOR	Mammalian target of rapamycin
NCDB	National Cancer Database
NGS	Next-generation sequencing
NRAS	Neuroblastoma RAS viral oncogene homolog
NSCLC	Non-small cell lung cancer
OR	Odds ratio
ORR	Objective response rate
OS	Overall survival
PCR	Polymerase chain reaction
PD-L1	Programmed death-ligand 1
PFS	Progression-free survival
PI3K	Phosphoinositide 3-kinase
PIK3CA	Phosphatidylinositol-4,5-bisphosphate 3-kinase catalytic subunit alpha
PTEN	Phosphatase and tensin homolog
RAS	Rat sarcoma virus
RFLP	Restriction fragment length polymorphism
RR	Risk ratio
SCC	Squamous cell carcinoma
TKD	Tyrosine kinase domain
TKI	Tyrosine kinase inhibitor
TMB	Tumor mutational burden
TNBC	Triple-negative breast cancer
TP53	Tumor protein p53

## References

[1] Cicenas J., Zalyte E., Bairoch A., Gaudet P. (2018). Kinases and Cancer. Cancers.

[2] Colicelli J. (2004). Human RAS superfamily proteins and related GTPases. Sci. STKE.

[3] Pai E.F., Krengel U., Petsko G.A., Goody R.S., Kabsch W., Wittinghofer A. (1990). Refined crystal structure of the triphosphate conformation of H-ras p21 at 1.35 Å resolution: Implications for the mechanism of GTP hydrolysis. EMBO J..

[4] Du T., Zhao H. (2022). Texture analysis for EGFR mutation prediction in breast cancer. Cancer Res..

[5] Albadari N., Li S. (2024). Deciphering treatment resistance in metastatic colorectal cancer: Roles of drug transports, EGFR mutations, and HGF/c-MET signaling. Front. Pharmacol..

[6] Ali M.A., Zahra O.S., Morsi M.I., El Safwany M.M., El Feky S.E. (2024). Predictive role of [^18^F]FDG PET-CT radiomic parameters for KRAS/BRAF/EGFR mutations in metastatic colorectal cancer patients. EJNMMI Rep..

[7] Jeon Y.H., Lim A., Choi M.K., Choi H.S., Lee Y., Song H., Cho H.S., Cho J., Choi Y.L. (2026). Clinical and Molecular Characterization of a Rare EGFR cis Compound L833V/H835L Mutation in Non-Small Cell Lung Cancer. Cancer Res. Commun..

[8] Braschel C., Fabikan H., Rodriguez V.M., Hochmair M.J., Illini O., Ay L., Weinlinger C., Krainer-Jacobs J., Müser N., Valipour A. (2026). Clinical Impact of EGFR Mutation Subtypes on Treatment Outcomes in Advanced Non-Small Cell Lung Cancer: An Austrian Real-World Study. Cancers.

[9] Lin S.H., Kahangire D.A., Nagar S.P., Ahn M.J., Affi R., Agulnik J., Shih J.Y., Hochmair M.J., Tufman A., Debieuvre D. (2026). EGFR mutation status, treatment patterns, and outcomes in resectable early-stage non-small cell lung cancer prior to adjuvant EGFR-TKI approval: An international real-world study. Lung Cancer.

[10] Shah A.A., Mani A., Akhtar S. (2026). Tumor Angiogenesis and EGFR-Mutated Cancers: Structural Insights, Mutation Dynamics, and Innovative Therapeutic Strategies. Curr. Top. Med. Chem..

[11] Yang T., Xu Y., Lin X., Chen G., Wang F. (2026). Metastatic breast cancer in primary lung cancer with compound EGFR mutations: A case report. Oncol. Lett..

[12] Yang Y., Zhang N., Shi L., Chen Z., Lu B., Liu Z. (2026). Analysis of Acquired Fusion Mutations in EGFR-TKIs-Resistant Patients with Advanced Lung Cancer. J. Coll. Physicians Surg. Pak..

[13] Kropf-Sanchen C., Frost N., Kuon J., Wermke M., Krüger S., Fuchs F., Wiesweg M., Christopoulos P., Thomas M., Gaisa N.T. (2026). RET fusions as a resistance mechanism to EGFR-TKIs in EGFR-mutant NSCLC: A real-world multicenter analysis. Clin. Lung Cancer.

[14] Gow C.H., Hsieh M.S., Chen Y.L., Liu Y.N., Wu S.G., Shih J.Y. (2023). Survival outcomes and prognostic factors of lung cancer patients with the MET exon 14 skipping mutation: A single-center real-world study. Front. Oncol..

[15] Ding C., Qiu Y., Zhang J., Wei W., Gao H., Yuan Y., Wang X. (2023). Clinicopathological characteristics of Non-Small Cell Lung Cancer (NSCLC) patients with c-MET exon 14 skipping mutation, MET overexpression and amplification. BMC Pulm. Med..

[16] Charbel C., Causa Andrieu P.I., Soliman M., Woo S., Zheng J., Capanu M., Nikolovski I., Vargas H.A., Abusamra M., Carlo M.I. (2024). The Prevalence and Radiologic Features of Renal Cancers Associated with FLCN, BAP1, SDH, and MET Germline Mutations. Radiol. Imaging Cancer.

[17] Guérin C., Tulasne D. (2024). Recording and classifying MET receptor mutations in cancers. eLife.

[18] Yang X.R., Zhong S.M., Jin Z.Y., Gào X., Wu Y., Zhou Q., Li Y.Q., Liu S.M., Wu Y.L. (2024). EGFR exon 20 insertion mutation and MET exon 14 skipping mutation in non-small cell lung cancer: A scoping review in the Chinese population. Transl. Lung Cancer Res..

[19] Dhiab R.B., Loyaux R., Garinet S., Bastide M., Léonard-Goyet S., Fabre E., Mansuet-Lupo A., Gibault L., Jouveshomme S., Giroux-Leprieur E. (2025). MET exon 14 skipping mutations in non-small-cell lung cancer a 3 years screening experience. Sci. Rep..

[20] Chen L., Li Y., Zhao H., Huang J., Yan H., Lin X., Zhao B. (2025). Pan-cancer analysis of MET mutation and its association with the efficacy of immune checkpoint blockade. Genes Dis..

[21] Liao J., Wu S., Min Y., Li Y., Nie Y., Chen Q., Mao Z., Zong Q., Gao N., Zhang D. (2025). The Landscape of MET Exon 14 Skipping Mutations in Patients with Lung Cancer Identified by Next-Generation Sequencing. JTO Clin. Res. Rep..

[22] Xue Q., Wang Y., Zheng Q., Huang Z., Lin Y., Jin Y., Li Y. (2025). MET Exon 14 Skipping Mutations in Lung Cancer: Clinical-Pathological Characteristics and Immune Microenvironment. Curr. Oncol..

[23] Zhang Y., Luo N., Ge M., Zhang Y., Chen D., Li Y. (2026). The Mutation Patterns of MET Gene in Lung Cancer and Brain Tumors: Clinical and Therapeutic Implications. Cancer Med..

[24] Kashizaki F., Oouchi M., Watanabe S., Shonai S., Orii R., Takeuchi K., Konishi K., Suematsu N. (2026). Lung cancer with MET exon 14 skipping mutation presenting with a crazy-paving appearance. Am. J. Respir. Crit. Care Med..

[25] Ger M., Kaupinis A., Petrulionis M., Kurlinkius B., Cicenas J., Sileikis A., Valius M., Strupas K. (2018). Proteomic Identification of FLT3 and PCBP3 as Potential Prognostic Biomarkers for Pancreatic Cancer. Anticancer Res..

[26] Aung N.E.E., Panyasai S., Kongpan C., Pornprasert S. (2022). FLT3 Gene Mutations in Acute Myeloid Leukemia Patients in Northeast Thailand. Med. Sci. Monit. Basic Res..

[27] Moualla Y., Alachkar A., Alhalabi R., Moassass F., Al-Halabi B., Darwish A., Al-Achkar W. (2022). Evaluating the clinical significance of FLT3 mutation status in Syrian newly diagnosed acute myeloid leukemia patients with normal karyotype. Heliyon.

[28] Koo M., Cho B.S., Park G., Yoon S., Kim Y., Kim D., Lee S.E., Byun J.M., Kim D.Y., Lee J.H. (2023). Prognostic value of the mutation types and dynamics of FLT3-ITD in acute myeloid leukemia. Eur. J. Haematol..

[29] Tarlock K., Gerbing R.B., Ries R.E., Alonzo T.A., Wang J., Leonti A., Zamora A., Brodersen L.E., Zhang Q., Chen L. (2024). Prognostic impact of cooccurring mutations in FLT3-ITD pediatric acute myeloid leukemia. Blood Adv..

[30] Arwanih E.Y., Louisa M., Sari R.M., Satria A.R., Suraya R., Kosasih A.S., Fauzi A.R., Ikhsan M.R., Rinaldi I. (2024). Identification of a novel mutation of the FLT3 gene located on the juxtamembrane domain from acute myeloid leukemia patients. Mol. Biol. Rep..

[31] Rattanathammethee T., Rattarittamrong E., Wanitpongpun C., Kungwankiattichai S., Owattanapanich W., Chanswangphuwana C., Polprasert C., Piyajaroenkij T., Niparuck P., Saengboon S. (2025). Real-world data on adult AML with FLT3-ITD mutation from the Thai acute leukemia working group. Front. Oncol..

[32] Pospiech M., Ngo M.P., Alrawashdeh M., Qamar U., Ali A., Tam E., Yaghmour G., Alachkar H., Ladha A. (2026). Acute Myeloid Leukemia with a Non-Canonical FLT3 V491L Mutation: A Case Report with Ex Vivo FLT3 Inhibitors Sensitivity Testing. J. Med. Cases.

[33] Chang Y.S., Tsai F.M., Wu Y.W., Yang C.Y., Tsai X.C.H., Ni S.C., Lo M.Y., Lee W.H., Lin C.C., Kuo Y.Y. (2026). Prognostic impact of co-occurring FLT3 mutations across molecular subgroups in intensively treated acute myeloid leukemia: Insights from real-world genomic data. Blood Cancer J..

[34] Cicenas J., Tamosaitis L., Kvederaviciute K., Tarvydas R., Staniute G., Kalyan K., Meskinyte-Kausiliene E., Stankevicius V., Valius M. (2017). KRAS, NRAS and BRAF mutations in colorectal cancer and melanoma. Med. Oncol..

[35] Wang L., Lu Q., Jiang K., Hong R., Wang S., Xu F. (2021). BRAF V600E Mutation in Triple-Negative Breast Cancer: A Case Report and Literature Review. Oncol. Res. Treat..

[36] Jafarian A., Jafaripour M., Gharib M., Salehi M., Mohamadian Roshan N., Etemad S., Mirshekar K., Sheikhi M., Heidari M., Ahmadian B. (2023). Molecular Status of BRAF Mutation in Epithelial Ovarian Cancer: An Analysis of 57 Cases in the Northeast of Iran. Iran. J. Pathol..

[37] Arons E., Tai C.H., Suraj J., Liu Y., Day C.P., Raffeld M., Xi L., Zhou H., Gould M., Shpilman I. (2025). Non-V600E BRAF mutations and treatment for hairy cell leukemia. Blood.

[38] Al-Abdulmalek A., Landry E., Abdulgayoom M., Al-Mashdali A.F., Dalol A.A., Muthanna B., Routy J.P., Mohamed S.F. (2026). BRAF Alterations in Chronic Lymphocytic Leukemia: Genomic Landscape, Co-Mutation Patterns, and Clinical Relevance. Curr. Hematol. Malig. Rep..

[39] Berardi G.G., Muthanna J., Wang Y.L., Olszanski A.J. (2025). Recurrent Melanoma in a Patient with Chronic Lymphocytic Leukemia (CLL) Presenting with an Apparent Co-Existing NRAS and BRAF Mutation: A Diagnostic and Treatment Conundrum. Int. J. Mol. Sci..

[40] Yang W., Duan Y., Huang X., Song W., Qian J., Zeng Y., Guo T., Wu Y., Li H., Zou X. (2026). Distinct clinical and genomic profiles of BRAF mutation subtypes in Chinese colorectal cancer: A retrospective cohort study with cross-population validation. Cell. Oncol..

[41] Lu K., Shen J.P., Lopez-Diaz F.J., Leal A., Mambetsariev I., Parikh K., Hazim A., Woodward B.D., Madduri A., Khurshid F. (2025). Diversity of BRAF mutations in non-small cell lung cancer and implications on treatment. npj Precis. Oncol..

[42] Catani G., Kim S., Waisberg F., Enrico D., Luca R., Esteso F., Bruno L., Rodríguez A., Bortz M., Freile B. (2025). Patients with Colorectal Cancer and BRAFV600E-Mutation in Argentina: A Real-World Study—The EMOGI-CRC01 Study. Cancers.

[43] Ono Y., Numata K., Iguchi K., Uchiyama M., Asari M., Rino Y., Saito A., Shiozawa M. (2025). Clinicopathological Features and Prognosis of Unresectable Colorectal Cancer with the BRAF V600E Mutation. Cancer Diagn. Progn..

[44] Feng X., Zeng R., Lyu M., Chen X., Xu Z., Hu Y., Bao Z., Sun X., Zhao J., Zhou L. (2025). Clinical and molecular characteristics, therapeutic strategies, and prognosis of non-small cell lung cancer patients harboring primary and acquired BRAF mutations. Front. Oncol..

[45] El Sayed M., Youssef S., Shealy M.W., El Harati M. (2026). Prognostic and clinicopathologic significance of BRAF mutation in colon cancer by MSI status and stage: A large cohort analysis. Surg. Oncol..

[46] Rahme C., Josianne B., Viviane T.S., Joseph K. (2026). Prevalence of BRAF Mutation in Colorectal Cancer Among Lebanese Patients: A Descriptive Study. J. Clin. Med..

[47] Cicenas J., Kvederaviciute K., Meskinyte I., Meskinyte-Kausiliene E., Skeberdyte A., Cicenas J. (2017). KRAS, TP53, CDKN2A, SMAD4, BRCA1, and BRCA2 Mutations in Pancreatic Cancer. Cancers.

[48] Johnson H., Ali A., Zhang X., Wang T., Simoulis A., Wingren A.G., Persson J.L. (2022). K-RAS Associated Gene-Mutation-Based Algorithm for Prediction of Treatment Response of Patients with Subtypes of Breast Cancer and Especially Triple-Negative Cancer. Cancers.

[49] Xu Y., Fang H., Chen Y., Tang Y., Sun H., Kong Z., Yang F., Kirschner-Schwabe R., Zhu L., Toker A. (2022). The KRAS-G12D mutation induces metabolic vulnerability in B-cell acute lymphoblastic leukemia. iScience.

[50] Kamian S., Ashoori H., Vahidian F., Davoudi S. (2023). The Relevance of Common K-RAS Gene Mutations and K-RAS mRNA Expression with Clinicopathological Findings and Survival in Breast Cancer. Asian Pac. J. Cancer Prev..

[51] Aytac A., Demir B., Balcik O.Y., Tuzcu T.U., Oktay E., Erdogdu I.H., Tanriverdi O., Yazici O. (2025). KRAS mutation subtypes in metastatic non-small cell lung cancer. Am. J. Cancer Res..

[52] El Sayed M., Youssef S., Shealy M.W., El Harati M. (2026). RAS Mutation Status as a Prognostic Marker and Predictor of Therapy Response in Colorectal Cancer: An NCDB Analysis. J. Gastrointest. Cancer.

[53] Tjader N.P., Ramroop J., Gandhi T., Dauch C., Meadows O., Stevens P., Pearlman R., Hampel H., Aglago E.K., Berndt S.I. (2026). Association of germline variants with KRAS-mutation status in colorectal cancer. Sci. Rep..

[54] Noordhof A., Asmara O.D., de Jong K., Gijtenbeek R.G., Huitema Y.M., van Vollenhoven F.H., Venmans B.J., Hendriks L., van Geffen W.H. (2026). KRAS G12C inhibitors versus chemotherapy in second line and beyond in adults with advanced or metastatic non-small cell lung cancer (NSCLC) harbouring the KRAS G12C mutation. Cochrane Database Syst. Rev..

[55] Raji S., Zaribafzadeh H., Jones T., Kanu E., Tong K., Fletcher A., Howell T.C., McCall S.J., Marks J.R., Rogers B. (2026). Prognostic Implications of Codon-Specific KRAS Mutations in Localized and Advanced Stages of Pancreatic Cancer. JCO Precis. Oncol..

[56] Ishikawa S., Ouchi K., Wakayama S., Oyamada S., Iwasaki T., Numakura R., Yoshida Y., Taniguchi S., Kasahara Y., Komine K. (2026). Coexistence of TP53 and KRAS mutations identifies a molecular subset of biliary tract cancer with poor overall survival after first-line immunochemotherapy. Eur. J. Cancer.

[57] Reinhardt K., Stückrath K., Hartung C., Kaufhold S., Uleer C., Hanf V., Lantzsch T., Peschel S., John J., Pöhler M. (2022). PIK3CA-mutations in breast cancer. Breast Cancer Res. Treat..

[58] Bredin H.K., Krakstad C., Hoivik E.A. (2023). PIK3CA mutations and their impact on survival outcomes of patients with endometrial cancer: A systematic review and meta-analysis. PLoS ONE.

[59] Daher S., Zer A., Tschernichovsky R., Yacobi R., Barshack I., Tsabari S., Rottenberg Y., Zick A., Gottfried T., Lobachov A. (2023). Driver mutation characteristics of phosphatidylinositol-4,5-bisphosphate 3-kinase catalytic subunit alpha (PIK3CA) in advanced non-small cell lung cancer. Lung Cancer.

[60] Liu Y., Li H., Li X., Zhang T., Zhang Y., Zhu J., Cui H., Li R., Cheng Y. (2024). Highly consistency of PIK3CA mutation spectrum between circulating tumor DNA and paired tissue in lung cancer patients. Heliyon.

[61] Yasin F., Sokol E., Vasan N., Pavlick D.C., Huang R.S.P., Pelletier M., Levy M.A., Pusztai L., Lacy J., Zhang J.Y. (2024). Molecular characteristics of advanced colorectal cancer and multi-hit PIK3CA mutations. Oncologist.

[62] Shen Q., Tian C., Luo X., Yang F., Jiang P., Zheng Y. (2025). Somatic Mutations Are Associated with Lymph Node Metastasis in Endometrial Cancer. Sichuan Da Xue Xue Bao Yi Xue Ban.

[63] Ghosh A., Moorthy A. (2024). Prevalence and effect of PIK3CA H1047R somatic mutation among Indian head and neck cancer patients. Saudi J. Biol. Sci..

[64] Rasheed A.H.R.S., Sekar P.K.C., Sreevalsan A., Veerabathiran R., Anand V. (2026). Oncogenic impact of PIK3CA, KRAS, and PTEN mutations in cervical cancer among South Indian women. Adv. Clin. Exp. Med..

[65] (2026). Clinicopathological significance of common PIK3CA exon mutations in non-metastatic colorectal cancer. Future Oncol..

[66] Zhang Y., Tong S., Wan L. (2025). Two first-generation KRAS inhibitor safety profiles: A disproportionality analysis of individual reports from the FDA adverse event reporting system. Expert Opin. Drug Saf..

